# Menthol—A Local Anæsthetic

**Published:** 1886-02

**Authors:** 


### Menthol—A Local Anæsthetic.

It is proposed (*Progres Medical,* Sept. 12,) to substitute this in place of cocaine. This drug has been known to the Japanese for 200 years. It is formed in the essences of menthe made in Asia, and is obtained in crystals by evaporating these essences. It exists in large proportion in the volatile oil of *mentha arvensis.* The preparations of menthe quiet the excessive sensibility of the laryngo-bronchial mucous membranes. They suppress the pain but not the sensibility. The tickling and desire to cough disappear. In a word, it is an antalgic remedy. Recently, Rosenberg has proposed to substitute it for cocaine. Its effect lasts from a quarter of an hour to an hour, or even an hour and a half. It is used in solution, alcoholic and oleaginous, of 20—50 per cent. for the nasal mucous membrane, 10 per. cent. for the larynx, and weaker for the conjunctiva. It produces an anæsthesia very pronounced, and seems to act with more energy upon the ocular mucous membrane than upon the buccal or nasal. It contracts the blood-vessels.

